# Endourologic Intervention for Management of Infertility in a Man with Zinner Syndrome Resulting in a Natural Pregnancy

**DOI:** 10.1089/cren.2016.0010

**Published:** 2016-04-01

**Authors:** Ismaeel Aghaways, Shyaw M. Ahmed

**Affiliations:** Urology Unit, Department of Surgery, School of Medicine, University of Sulaimani, Sulaimani, Iraq.

## Abstract

***Background:*** Ipsilateral renal agenesis associated with seminal vesicular cysts is an uncommon finding. Zinner syndrome is a rare variant of wolffian duct anomalies with a triad of seminal vesicle cyst, ipsilateral renal agenesis, and male fertility problems due to ejaculatory duct obstruction (EDO).

***Case Presentation:*** A 28-year-old man with 6 years history of primary infertility presented with left-side lower abdominal pain. A palpable cystic mass was found on digital rectal examination. Semen analysis revealed low volume ejaculate azoospermia. Abdominal ultrasonography revealed a single right kidney and transrectal ultrasonography showed an evidence of left EDO. Transurethral resection of the ejaculatory duct was performed. Semen analysis after 2 weeks showed normal sperm count (23M) and acceptable progressive motility (24%). Eight weeks later, his wife was pregnant with a 7-week viable fetus.

***Conclusion:*** Although not a common disease, a careful physical examination and thorough semen analysis interpretation should guide clinicians to diagnose a surgically treatable syndromic cause of male infertility.

## Introduction and Background

Our patient presented with pain in his left lower part of his abdomen and inability to become a father for the past 6 years. After thorough history and examining the patient, we realized that his left kidney was absent and a block in his ejaculate (semen) path was making him infertile. He was managed by passing a resectoscope (camera with a resection loop) through his urethra and relieving the block of semen pathway. Two weeks later sperms appeared in his ejaculate and after 2 months his wife became pregnant with a single healthy fetus.

## Case Presentation

### History

A 28-year-old gentleman complained of left iliac fossa pain for nearly 1 month. Pain was referred to the left testis. There were no associated lower urinary tract symptoms, no pain during ejaculation, or perineal pain. His partner has no known cause of infertility. The patient had a 6 year history of primary infertility. In addition, he admitted to a history of a single episode of self-limiting hematospermia 7 years ago. The patient denied any history of sexual contact before marriage. There was no family history of infertility and he is a smoker with five pack-year.

### Physical examination

Our patient was a well looking, mentally fit, young man with normal secondary sexual characteristics. On genital examination, normal size testes with bilateral palpable vasa deferentia were identified, with left-side testis slightly larger than the right. There was no evidence of varicocele. On digital rectal examination, there was a cystic mass palpable above the prostate mostly on the left side (upper limit was not reachable), with normal prostate.

### Diagnosis

**FIG. 1.**
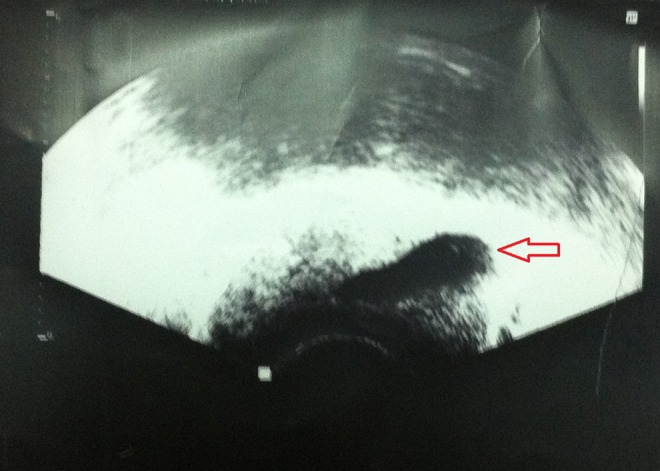
TRUS showing a tubular cystic structure inserting to the site of left ED associated with prominent terminal part of vas deferens (left EDO). ED, ejaculatory duct; EDO, ejaculatory duct obstruction; TRUS, transrectal ultrasonography.

Semen analysis revealed low volume, normal pH, and low fructose azoospermia. On two occasions, semen analysis has shown cryptozoospermia. Blood tests showed normal hormonal analysis. No abnormal finding was detected on scrotal ultrasonography. Abdominal ultrasonic examination detected nonobservable left kidney with evidence of dilated tubular structure (20 mm long) and (7 mm wide) seen extending from left seminal vesicle to the prostate. Transrectal ultrasonography (TRUS) (Fig. 1) showed evidence of tubular cystic structure inserting into the site of the ejaculatory duct (ED) on the left side associated with prominent terminal part of the vas deferens. The figure is suggestive of left-side ejaculatory duct obstruction (EDO).

### Intervention

**FIG. 2.**
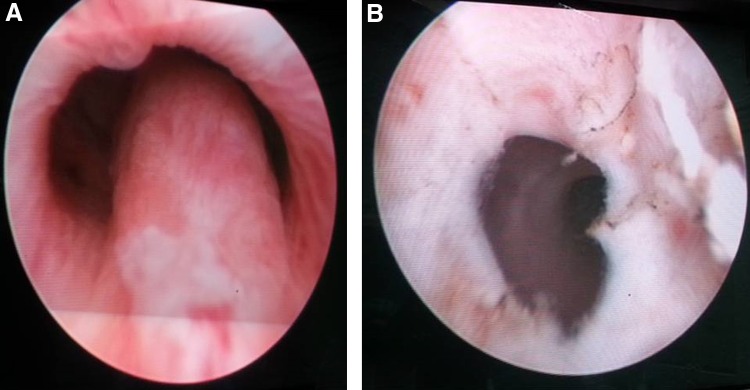
**(A)** Coronal section of a contrast-enhanced abdominal computed tomography showing single right kidney, left kidney agenesis, and right-sided seminal vesicular cystic dilatation. **(B)** Axial section single right kidney with evidence of compensated hypertrophy.

Under general anesthesia using rigid urethrocystoscopy, an optical urethrotomy was done for a stricture at bulbomembraneous urethra. Then a prominent verumontanum was identified at the prostatic urethra (Fig. 2A). Transuretral resection of the ejaculatory duct (TURED) was performed. After deroofing, a dilated seminal vesicle appeared from inside (Fig. 2B) with a brownish opaque fluid coming out. The patient was kept one night in the hospital without urethral catheter on analgesia and empirical antibiotics. He was then discharged next day on antibiotics for 5 days.

### Follow-up

**FIG. 3.**
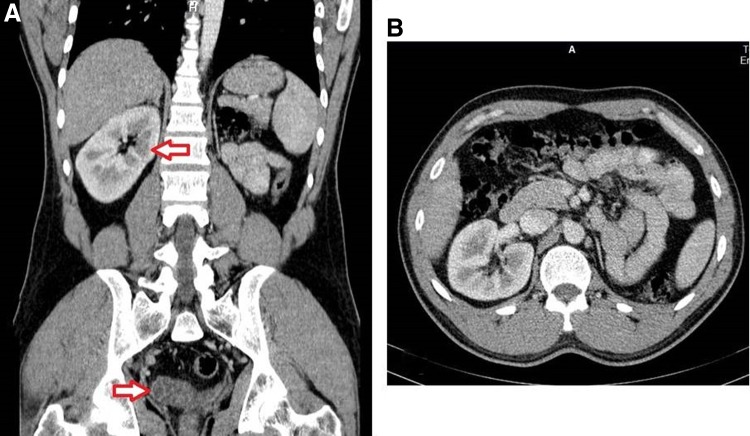
**(A)** Prominent verumontanum at prostatic urethra on rigid endoscopic examination (underlying EDO). **(B)** Post-TURED deroofing of verumontanum showing a large cavity with dilated seminal vesicular pockets. TURED, transuretral resection of the ejaculatory duct.

One week later, the patient presented with an attack of left-sided epididymitis and was treated with appropriate antibiotic and analgesia. However, it was only after the TURED there was a chance to send for a retrospective computed tomography (Fig. 3A), at least to confirm the renal agenesis and complete the triad of the syndrome, which revealed single right-sided kidney with compensated hypertrophy and nonobservable left kidney (agenesis). In addition, cystic enlargement of the right seminal vesicle was identified.

### Outcome

Two weeks post-TURED, semen analysis showed normal volume with 23 million sperm count and 24% progressive motility. Two months later, a B-hcG for his partner was positive. Obstetric ultrasonography showed a gravid uterus with a 7-week single healthy viable embryo and a positive fetal heart.

## Discussion

Ipsilateral renal agenesis associated with seminal vesicular cysts is an uncommon finding.^1^ Zinner syndrome is a variant of wolffian duct anomalies with a triad of seminal vesicle cyst, ipsilateral renal agenesis, and male fertility problems due to EDO.^2^ Abnormal distal mesonephric duct development resulting in faulty ureteral bud fusion with metanephros (renal agenesis) coupled with atretic EDs and abnormal seminal vesicular dilatation is a possible embryologic explanation for this urologic anomaly.^2^

Patients with Zinner syndrome usually have no symptoms at all or may present with pressure symptoms of cystic dilatation of seminal vesicles such as pelvic pain and painful ejaculation in their early 30s to late 40s.^1^ However, it is not uncommon for the patient to have infertility as a solitary complaint. As a result of a wide range symptomatology of this syndrome, late diagnosis of the infertility etiology and prompt management are frequent events.

Although our patient's semen analysis has lately revealed only azoospermia, previous tests showed occasional sperms after centrifugation of his samples. Nevertheless, he always had low volume ejaculate, which denotes an evidence of obstruction. Low fructose is an additional finding for partial EDO. Hence, awareness of this triad and proper semen analysis interpretation is an essential part of clinical diagnosis.

Different tools have been described for diagnosis in the literature, ranging from abdominal and pelvic ultrasonography, TRUS, and computed tomography.^3^ Our case has recently presented with left sided pelvic pain and also had a 6 year history of primary infertility; physical examination and review of previous investigations have led us to send him for abdominal ultrasonography and TRUS. We diagnosed left-side EDO and proceeded with TURED. Unfortunately, we retrospectively sent the patient for a computed tomography to confirm renal agenesis and diagnose the Zinner syndrome. However, post-TURED showed prominent right-sided seminal vesicular cyst possibly due to evacuation of the left side seminal vesicle content at the time of resection.

Management is obviously dependent on the main complaint of the patient. A number of treatment options have been described beginning with conservative management for asymptomatic cases. TURED will usually suffice for patients with EDO and infertility.^3^ Furthermore, formal laparoscopic or rather more sophisticated robotic assisted laparoscopic excision for large symptomatic cysts has also been described.^3^

## Conclusion

Zinner syndrome is not a common cause of male subfertility, though awareness of the condition when managing men with infertility through a careful physical examination and thorough semen analysis interpretation should guide clinicians to diagnose a surgically treatable syndromic cause of male infertility. A simple TURED seems sufficient to relieve the EDO, resume normal semen pathway, and regain fertility.

## Abbreviations Used

ED: ejaculatory duct
EDO: ejaculatory duct obstruction
TRUS: transrectal ultrasonography
TURED: transuretral resection of the ejaculatory duct
